# DEPDC1B collaborates with GABRD to regulate ESCC progression

**DOI:** 10.1186/s12935-022-02593-z

**Published:** 2022-06-15

**Authors:** Yunfeng Yuan, Wei Ping, Ruijie Zhang, Zhipeng Hao, Ni Zhang

**Affiliations:** 1 Department of Thoracic Surgery, Tongji Hospital, Tongji Medical College, Huazhong University of Science and Technology, No. 1095, Jiefang Avenue, Wuhan, 430030 Hubei China; 2 Department of Thoracic Surgery, Zhongshan Hospital, Fudan University, Shanghai, 200030 China

**Keywords:** ESCC, DEPDC1B, GABRD, Prognosis, Phenotype

## Abstract

**Background:**

Esophageal squamous cell carcinoma (ESCC) is the leading cause of cancer-related death worldwide with a poor prognosis. Given that DEPDC1B plays a key role in multiple cancers, the role of this molecule in ESCC was explored to identify potential targets for ESCC patients.

**Method:**

The expression level of DEPDC1B in ESCC was revealed based on the TCGA database and immunohistochemical experiments on clinical tissues. The correlation between DEPDC1B and survival of ESCC patients was analyzed by Kaplan–Meier method. Small hairpin RNA (shRNA)-mediated silencing of DEPDC1B expression in ESCC cells and performed a series of in vitro and in vivo functional validations.

**Result:**

DEPDC1B was overexpressed in ESCC. High expression of DEPDC1B was significantly negatively correlated with overall survival in patients with ESCC. Moreover, knockdown of DEPDC1B inhibited ESCC cell proliferation, clone formation, migration, tumor formation and promoted apoptosis. Furthermore, knockdown of DEPDC1B leaded to significant downregulation of GABRD in ESCC cells. Meanwhile, GABRD expression was upregulated in ESCC, and its silencing can inhibit the proliferation and migration of the tumor cells. Interestingly, there was a protein interaction between DEPDC1B and GABRD. Functionally, GABRD knockdown partially reversed the contribution of DEPDC1B to ESCC progression. In addition, GABRD regulated ESCC progression may depend on PI3K/AKT/mTOR signaling pathway.

**Conclusion:**

DEPDC1B collaborated with GABRD to regulate ESCC progression, and inhibition of this signaling axis may be a potential therapeutic target for ESCC.

**Supplementary Information:**

The online version contains supplementary material available at 10.1186/s12935-022-02593-z.

## Introduction

Esophageal carcinoma (ESCA) is a common malignancy with increasing incidence [1]. Esophageal squamous cell carcinoma (ESCC) is the main subtype of ESCA and accounts for more than 90% of all cases [2]. In addition, ESCC is one of the most aggressive forms of human squamous cell carcinoma characterized by late-stage diagnosis, metastasis, frequent recurrence and therapy resistance [3]. Although advances in diagnostic and therapeutic techniques in recent decades have ameliorated the suffering of ESCC patients, the 5-year survival remains poor, estimated at 15–20% [4]. Recently, several novel therapies such as targeted therapy of epidermal growth factor receptor (EGFR) [5, 6], immunotherapy of PD-L1 [7, 8] were applied for treating the ESCC patient. However, clinical management of ESCC remains challenging, and the disease currently lacks effective targeted therapies [3]. Thus, identification of effective molecular therapeutic target for ESCC is an urgent priority.

DISHEVELLED, EGL-10, PLECKSTRIN (DEP) domain-containing 1B (DEPDC1B) and its paralog DEPDC1A are responsible for cell cycle regulation [9]. The *DEPDC1B* gene, localized at human chromosome 5q12, encodes a 61 kDa protein of 529 amino acids [10]. DEPDC1B consists of an N-terminal DEP domain and a C-terminal RHO-GAP (GTPase-activating protein)-like domain [11, 12]. The DEP domain is a globular region detected in DISHEVELLED, EGL-10 and PLECKSTRIN and contributes to the membrane localization [13]. Similarly, DEPDC1B is often membrane-related and is highly expressed during G2/M phase of the cell cycle [14]. The RHO-GAP domain is involved in RHO GTPase signaling such as RAC, CDC42 and RHO that regulates cell motility, growth, differentiation, cytoskeleton reorganization and cell cycle progression [15]. Related investigations reported that DEPDC1B is overexpressed in various cancer types, such as breast cancer, oral cancer, non-small-cell lung cancer, melanoma cancer, bladder cancer and prostate cancer [11, 12, 14, 16–20]. Overexpressed DEPDC1B contributes to the progression of hepatocellular carcinoma by CDK1 [21]. In addition, DEPDC1B regulates human chordoma progression through UBE2T-mediated ubiquitination of BIRC5 [22]. Numerous evidence suggested that DEPDC1B plays a key role in cancers, which may be a potential biomarker and therapeutic target. Thus, the biological function and clinical significance of DEPDC1B in ESCC was worth exploring.

In this study, the expression level of DEPDC1B in ESCC was revealed based on the TCGA database and immunohistochemical experiments on clinical tissues. The correlation between DEPDC1B and survival of ESCC patients was analyzed by Kaplan–Meier method. Furthermore, small hairpin RNA (shRNA)-mediated silencing of DEPDC1B expression in ESCC cells and performed a series of functional validations. Taken together, we explored the role of DEPDC1B in ESCC from clinical, in vitro, and in vivo levels.

## Methods

### Tissue microarray and immunohistochemical (IHC)

The study was approved by the ethical committee of Tongji Medical College, Huazhong University of Science and Technology. Tissue microarrays were prepared according to the methods provided in the literature [23], including tumor tissues and corresponding normal tissues of ESCC patients. Related patient information and written informed consents were collected. For IHC analysis, tissue microarrays were deparaffinized, rehydrated, blocked and incubated with primary and corresponding secondary antibodies. Notably, antibody information was listed in Additional file 1: Table S1. Diaminobenzene and hematoxylin were used for DAB color reaction. Staining slides were photographed with microscopy and observed by ImageScope and CaseViewer. All tissues were scored on the basis of percent staining and color intensity and were independently analyzed by two pathologists. Staining percentage ranks were categorized as: 1 (1–24%), 2 (25–49%), 3 (50–74%) and 4 (75–100%). Staining intensity were graded 0 (weak) to 3 (strong brown).

### Cell and chemicals

TE-4, TE-10, Eca-109 and TE-1 cell lines were purchased from the Cell Bank of Type Culture Collection of Chinese Academy of Science. All the cells were cultivated in RPMI-1640 medium (Gibco, USA) with 10% fetal bovine serum (FBS, Gibco, USA) under a humidified incubator at 37 °C with 5% CO_2_. AKT activator SC79 (Beyotime) was applied at the final concentration 5 μM.

### Cell transfection

Small hairpin RNA (shRNA) sequences targeting human DEPDC1B, CDK6 and GABRD were designed and inserted into lentivirus vector BR-V-108. The sequences were listed in Additional file 1: Table S2. Meanwhile, DEPDC1B was amplified and cloned into the lentivirus vector BR-V112 for DEPDC1B overexpression. Subsequently, recombinant lentivirus was transfected to Eca-109 and/or TE-1 cells using Lipofectamine 2000 (Thermo, USA) according to manufacturer’s instructions. Transfection efficiency was determined by green fluorescent protein signal at 72 h post-transfection.

### RNA extraction and qRT-PCR

Eca-109 and TE-1 cells were lysed and total RNA was isolated using TRIzol reagent (Thermo, USA). RNA concentration was determined using Nanodrop 2000/2000C spectrometer (Thermo, USA). The cDNA was reversely transcribed from RNA using M-MLV Kit (Promega, USA) and qRT-PCR was performed with SYBR Green mastermixs Kit (Vazyme) and Biosystems 7500 Sequence Detection system. GAPDH was used as inner control, and the primers used for the PCR reaction were shown in Additional file 1: Table S3. The relative quantitative data of gene expression were analyzed by the 2^−ΔΔCt^ method.

### Western blotting and co-immunoprecipitation (Co-IP)

Eca-109 and TE-1 cells were lysed in ice-cold RIPA buffer (Millipore), and collected protein. Protein concentration was quantified using a BCA Protein Assay Kit (HyClone-Pierce). The 20 μg protein per lane was loaded and separated using 10% sodium dodecyl sulfate polyacrylamide gel electrophoresis (Invitrogen, USA), and transferred onto polyvinylidene difluoride (PVDF) membranes in ice. Next, the membranes were blocked by 5% non-fat milk at room temperature for an hour. Subsequently, membranes were incubated with primary antibodies at 4 °C overnight followed by incubation of secondary antibody HRP goat anti-rabbit/mouse IgG for 2 h at room temperature. The blots were visualized by enhanced chemiluminescence (Amersham).

Co-IP assay was applied for identifying whether DEPDC1B physically interacted with GABRD in Eca-109 cells. 1.0 mg total proteins were incubated with anti-GABRD at 4 °C for 2 h. After centrifugation at 2000 × for 1 min, supernatant was discarded, and protein A/G beads were denatured in IP lysate buffer and 5 × loading buffer at 100 °C for 5 min. Finally, 20 μg protein sample was subjected to WB analysis as described above. Notably, the antibodies used in western blotting and Co-IP were listed in Additional file 1: Table S1.

### Celigo cell count assay

Eca-109 and TE-1 cells were seed into 96-well plates (2000 cells/well) and cultured in RPMI-1640 medium with 10% FBS at 37 °C with 5% CO_2_ for 5 days. The medium was changed every 3 days. Celigo image cytometer (Nexcelom Bioscience) was applied for cell count at 1–5 days and cell proliferation curve was graphed.

### Colony formation assay

Eca-109 and TE-1 cells were seeded into six-well plates (1000 cells/well) in triplicate and further cultured for 8 days. Of note, cells number more than 50 or cell size between 0.3 and 1.0 mm were considered as a single clone. Subsequently, all colonies were fixed by 4% paraformaldehyde, stained by Giemsa, and photographed with a digital camera. Colony forming rate equals colony number/inoculated cell number × 100%.

### Wound-healing assay

Eca-109 and TE-1 cells were collected and seeded into 96-well plates with a density of 50,000 cells/well in 100 μL medium and cultured at 37 °C with 5% CO_2_. Until cell grew more than 90% confluence, a scratch was made using a scratch tester paralleled the center of the lower end of the 96-well plate. Cells were washed with PBS twice and cultured in 0.5% PBS with 5% CO_2_ at 37 °C. Photographs were captured by Cellomics (ArrayScan VT1, Thermo) at the indicated time points and analyzed the migration area with Cellomics.

### Transwell assay

Corning Transwell Kit (Corning) was applied for transwell assay. After transfection, Eca-109 and TE-1 cells were collected, counted and incubated in the upper chamber with 100 μL RPMI-1640 medium without FBS in a 24-well plate (1 × 10^5^ cells/well). 600 μL medium supplemented with 30% FBS was added in the lower chamber. After incubation at 37 °C with 5% CO_2_, nonmetastatic cells were removed with a cotton swab. 400 μL Giemsa was added for staining and the migratory ability of cells was analyzed. The representative images were selected from 3 independent experiments, of which 5 fields per chamber were randomly selected for counting the cell numbers. Magnification was 200 × .

### Flow cytometry

Eca-109 and TE-1 cells were seeded into 6-well plates in triplicate and cultured for another 5 days. Next, the cells were harvested and washed with 4 °C ice-cold Hanks. After centrifugation at 1000 g, cells were resuspended in binding buffer and then stained in the dark by adding 5 μL of Annexin V-APC (eBioscience). Finally, apoptosis analysis was measured using FACSCalibur (BD Biosciences).

### Human apoptosis antibody assay

For human apoptosis antibody assay (Abcam), related proteins in human apoptosis signaling pathway were detected according to manufacturer’s instructions. Eca-109 cells protein were harvested and quantified as described above. Antibody arrays were incubated with protein (0.5 mg/mL) overnight at 4 °C followed by cocktail of biotin-conjugated antibodies overnight at 4 °C. Next, arrays were incubated with labelled streptavidin for 2 h. Enhanced chemiluminescence was used for visualizing and spots gray value was analyzed by Image J.

### Tumorigenicity mice model

Female BALB/c nude mice were purchased from Beijing Vital River Laboratory Animal Technology Co., Ltd. For tumorigenicity, 5 × 10^6^ lentivirus (shCtrl or shDEPDC1B) transfected Eca-109 cells were subcutaneously injected into each mouse (4-week-old, n = 10 per group). On the last day of feeding, the mice were anaesthetized by intraperitoneal injection of 0.7% pentobarbital sodium at a dose of 10 μL/g and after 10 min the nude mice were placed in a flat-box followed by cervical vertebrae. Tumor weight and tumor size were recorded after sacrificing mice and the tumor volume was calculated as π/6 × L × W^2^ (W, width at the widest point; L, perpendicular width). Finally, the tumor burden was assessed by in vivo imaging with the small animal multi-spectral living imaging system (Berthold Technologies). All animal experiments conformed to the European Parliament Directive (2010/63/EU) and were approved by the Institutional Animal Care and Use Committee at Tongji Medical College, Huazhong University of Science and Technology.

### RNA sequencing

Total RNA was extracted from Eca-109 cells (shCtrl or shDEPDC1B) using TRIzol. The quantity and quality of RNA were evaluated with a Nanodrop 2000 (Thermo). RIN value was evaluated with Agilent 2100 and Agilent RNA 6000 Nano Kit. Affymetrix PrimeView Human Gene Expression Arrays and Affymetrix Scanner 3000 (Affymetrix) were utilized for microarray analysis to obtain gene expression profiles according to the manufacturer’s instructions. Differentially expressed genes (DEGs) were selected based on P < 0.05 and fold change > 1.3. Bioinformatic analysis of DEGs based on Ingenuity Pathway Analysis (IPA) (Qiagen) was performed, and |Z-score|> 2 is considered meaningful.

### Statistical analysis

Data from independent experiments are shown as the mean ± standard deviations (SD). Statistical analysis between two groups was performed by Student’s *t*-test (two-tailed). DEPDC1B expression between ESCC tissues and normal tissues was analyzed with Rank Sum test analysis. The relationship of DEPDC1B expression and tumor characteristics in ESCC patients were analyzed with Mann–Whitney U analysis. Survival data were evaluated by using Kaplan–Meier survival analysis. P < 0.05 was considered as statistically significant difference.

## Results

### DEPDC1B is overexpressed in ESCC and correlates with poor prognosis

**Fig. 1:**
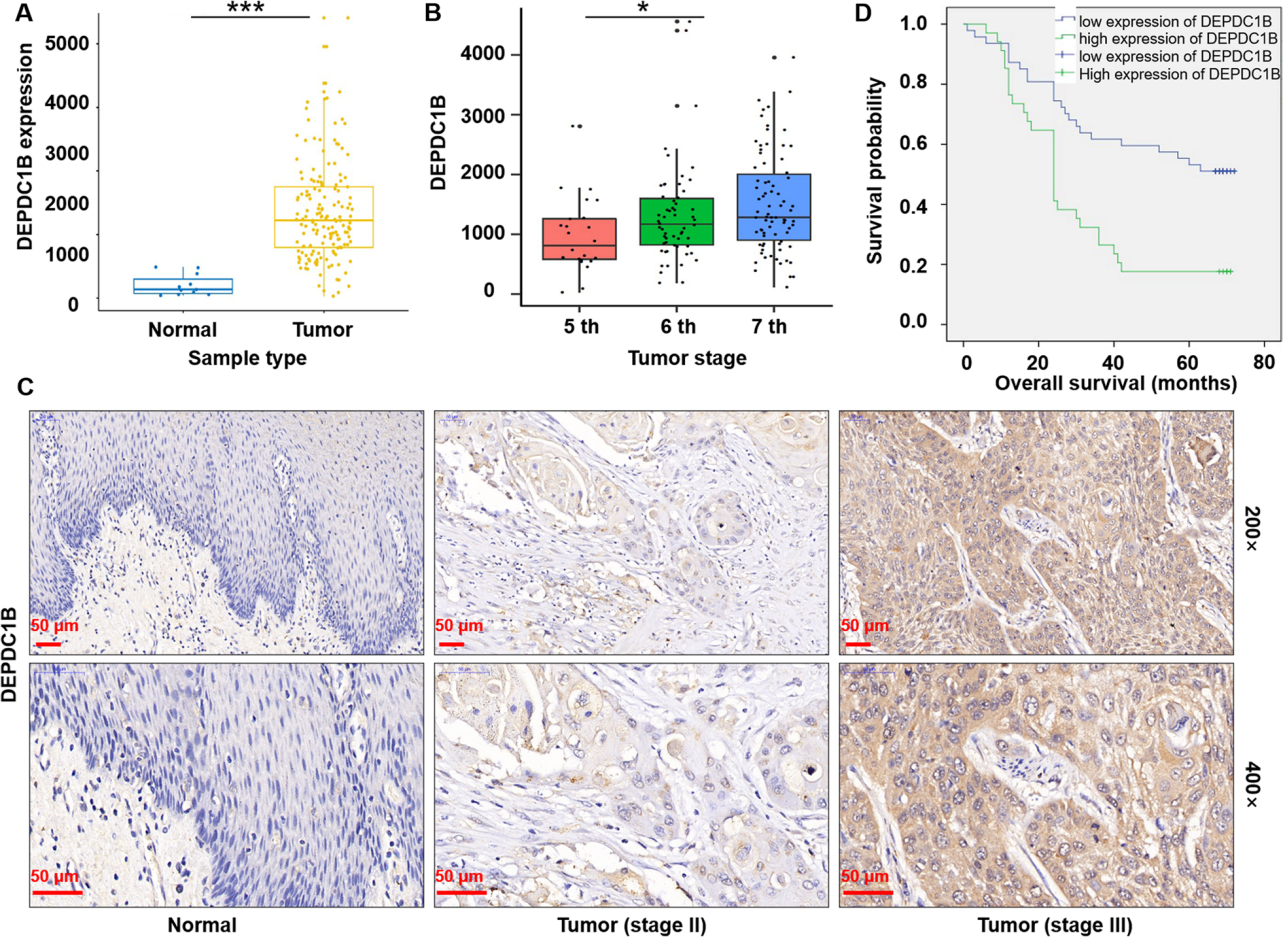
DEPDC1B is overexpressed in ESCC and correlates with poor prognosis. **A** Expression of DEPDC1B was performed based on the 161 tumor and 11 normal samples in the Cancer Genome Atlas (TCGA) database. **B** The correlation between DEPDC1B expression level and tumor stage was analyzed based on TCGA-ESCA database. **C** The expression level of DEPDC1B in ESCC was verified based on the tissue microarrays (tumor, n = 266; normal, n = 39) from clinical ESCC patients using immunohistochemical (IHC). **D** The correlation between DEPDC1B and survival of ESCC patients (n = 266) was analyzed by Kaplan–Meier method

Firstly, expression of DEPDC1B was performed based on the 161 tumor and 11 normal samples in the Cancer Genome Atlas (TCGA) database. As showed in Fig. 1A, DEPDC1B expression in tumor samples was significantly higher than that in normal samples (p < 0.001). Meanwhile, the prognostic analysis was performed based on the clinical information of TCGA-ESCA samples, suggested that the expression of DEPDC1B was significantly correlated with tumor stage (p < 0.05, Fig. 1B). Subsequently, the expression level of DEPDC1B in ESCC was verified based on the tissue microarrays (tumor, n = 266; normal, n = 39) from clinical ESCC patients using immunohistochemical experiments. As expected, the signal intensity of DEPDC1B in tumor tissue is stronger than that in normal tissue (Fig. 1C). According to IHC scores, DEPDC1B was highly expressed in 51.9% of tumor tissues but not in normal tissues (p < 0.001, Table 1). Furthermore, the correlation of DEPDC1B expression and tumor characteristics in ESCC patients were analyzed with Mann–Whitney U analysis. The results indicated that DEPDC1B expression was positively correlated with tumor infiltrate, lymphatic metastasis and stage in patients with ESCC (p < 0.001, Table 2). In addition, the correlation between DEPDC1B and survival of ESCC patients was analyzed by Kaplan–Meier method. We found that the high DEPDC1B expression was correlated with low survival rate of ESCC patients (p < 0.001, Fig. 1D). Overall, DEPDC1B expression was upregulated in ESCC and correlated with poor prognosis, which may serve as a possible diagnostic and prognostic marker.

**Table 1:** Expression patterns in esophagus cancer tissues and normal tissues revealed in immunohistochemistry analysis

DEPDC1B expression	Tumor tissue		Normal tissue		P value
	Cases	Percentage	Cases	Percentage	
Low	128	48.10%	39	100%	< 0.001
High	138	51.90%	0	–

**Table 2:** Expression patterns in esophagus cancer tissues and normal tissues revealed in immunohistochemistry analysis

Features	No. of patients	DEPDC1B expression		P value
		low	high	
All patients	266	128	138	
Age (years)				0.696
≤58	138	68	70	
>58	128	60	68	
Gender				0.946
Male	199	96	103	
Female	67	32	35	
Tumor size				0.16
≤4cm	57	34	23	
>4cm	50	23	27	
tumor infiltrate				< 0.01
T0	1	1	0	
T1	8	5	3	
T2	54	33	21	
T3	159	72	87	
T4	4	0	4	
Lymphatic metastasis (N)				< 0.01
N0	121	73	48	
N1	79	28	51	
N2	23	10	13	
N3	3	0	3	
Stage				< 0.001
0	1	1	0	
I	21	18	3	
II	112	59	53	
III	86	31	55	

### Silencing DEPDC1B inhibits cell proliferation and migration of ESCC

**Fig. 2:**
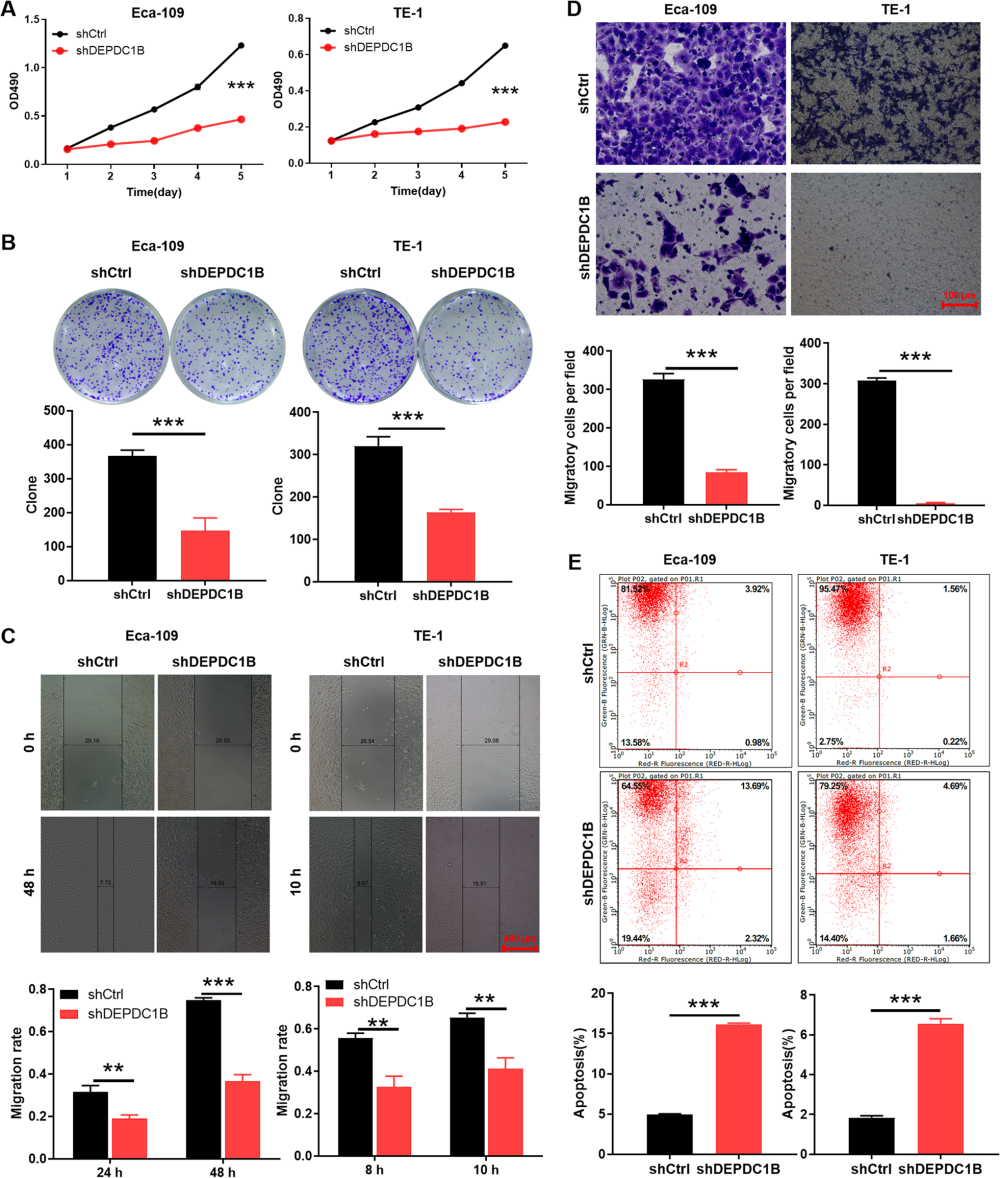
Silencing DEPDC1B inhibits ESCC cell proliferation, migration and promotes apoptosis. **A** The effects of DEPDC1B knockdown on Eca-109 and TE-1 cell proliferation in MTT assay. **B** Colony formation assay was used for evaluate the ability of ESCC cells to form colonies in shCtrl and shDEPDC1B groups. **C**, **D** wound-healing assay (**C**) and transwell assay (**D**) were used for determining migration of cells in shCtrl and shDEPDC1B groups. **E** The cell apoptosis was examined by flow cytometry upon DEPDC1B knockdown. The representative images were selected from 3 independent experiments, of which 5 fields were randomly selected for counting the cell numbers. **P < 0.01, ***P < 0.001

Furthermore, a series of functional validations were performed to explored the biological function of DEPDC1B in ESCC cells. Firstly, we silenced the expression of DEPDC1B in ESCC cell lines Eca-109 and TE-1 using shRNA-mediated interference. As shown in S1A, the knockdown effect of the shDEPDC1B-1 (shRNA targeting DEPDC1B) sequence was significantly higher than that of the other two sequences, which were used for cell transfection (p < 0.05). After transfecting cells with shCtrl (empty vector, negative control) and shDEPDC1B and detecting the mRNA and protein expression of DEPDC1B, the results showed that the expression of DEPDC1B was down-regulated in Eca-109 and TE-1 (p < 0.01, Additional file 1: Fig. S1A, B). Subsequently, the viability and colony forming ability of Eca-109 and TE-1 cells were assessed, respectively. The results indicated that the cell viability of shDEPDC1B group was significantly weaker than that of shCtrl (p < 0.001, Fig. 2A). Consistently, the shDEPDC1B group produced fewer and smaller cell clones compared to shCtrl (p < 0.001, Fig. 2B). In addition, wound-healing assay and transwell assay were used to assess the effect of DEPDC1B silencing on cell migration. Not surprisingly, knockdown of DEPDC1B significantly inhibits migration of Eca-109 and TE-1 cells (p < 0.001, Fig. 2C, D). Taken together, DEPDC1B contributed to proliferation and migration of ESCC cells in vitro.

### Silencing DEPDC1B enhances cell apoptosis of ESCC

Subsequently, the apoptotic capacity of Eca-109 and TE-1 cells (shCtrl vs shDEPDC1B) was assessed by flow cytometry. Obviously, ESCC cells in the DEPDC1B knockdown group had a higher rate of apoptosis than cells in the control group (p < 0.001, Fig. 2E). Moreover, the effect of DEPDC1B knockdown on the expression of canonical apoptosis-related proteins in Eca-109 cells was initially estimated. As illustrated in Additional file 1: Fig. S1D, DEPDC1B silencing downregulated the protein expression of HSP70, Survivin, and TNF-α, and upregulated the protein expression of CD40 and DR6 (p < 0.05). These data suggested that knockdown of DEPDC1B promoted apoptosis of ESCC cells, accompanied by abnormal expression of apoptosis-related proteins.

### Silencing DEPDC1B impairs tumorigenesis of ESCC cells

**Fig. 3:**
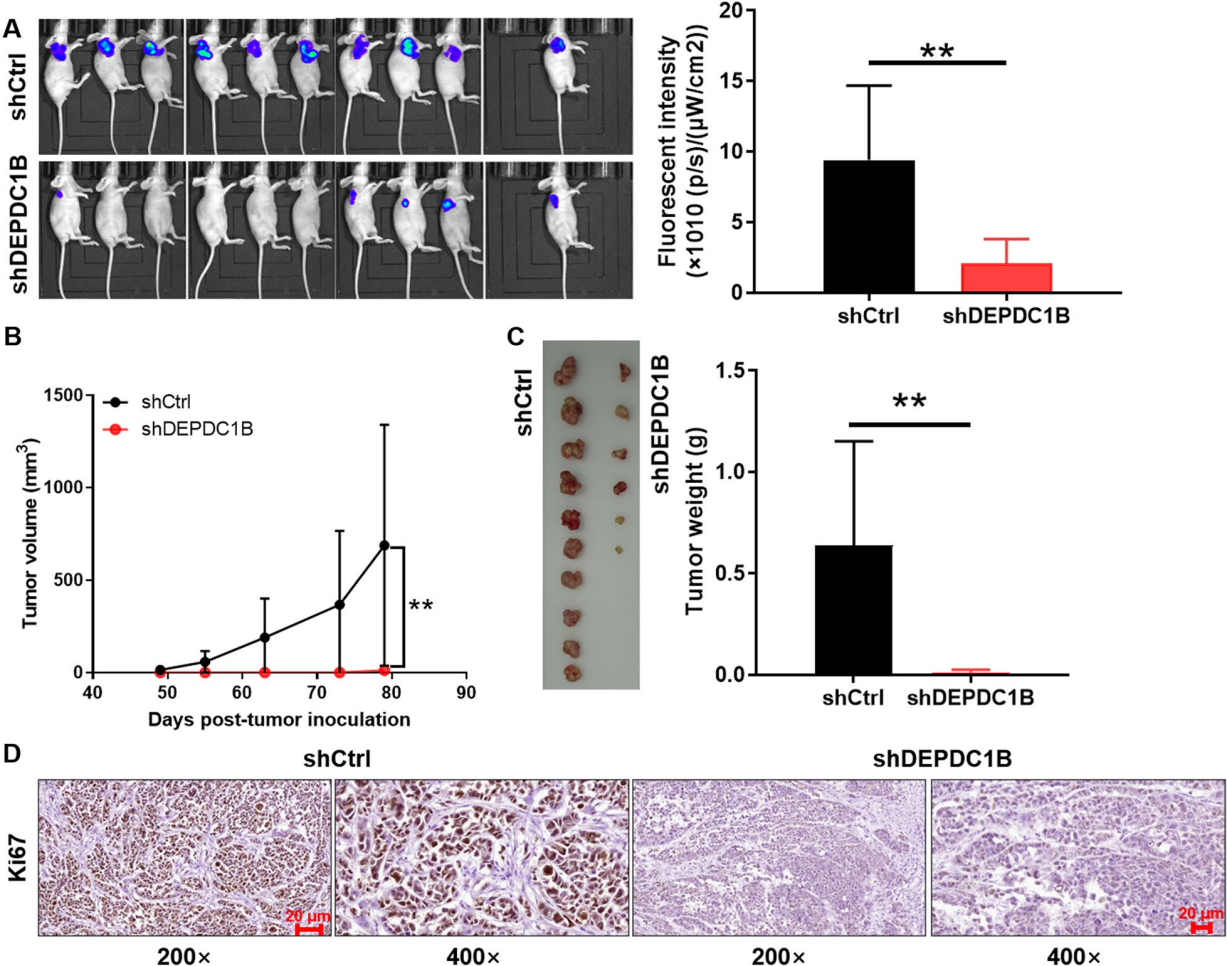
DEPDC1B is crucial for tumor growth in vivo. **A** A nude mice model of DEPDC1B knockdown through injecting Eca-109 cells was constructed and tumor burden in each group of mice was estimated by fluorescence intensity. **B** The volume of tumors was tested until sacrifice. **C** The weight of tumors was measured and photograph of tumors was taken after removing tumors. **D** The value of Ki-67 was detected by IHC in tumor sections. **P < 0.01

According to above in vitro loss-of-function experiments, we speculated that DEPDC1B played essential roles in ESCC tumor growth in vivo. Thus, xenograft mice model was established by subcutaneous injection of Eca-109 cells with or without DEPDC1B expression. The tumor growth curve and the observations of in vivo fluorescent imaging showed that tumors in shCtrl group grew more rapidly than those of shDEPDC1B group (p < 0.01, Fig. 3A, B). The smaller tumor size and weight in shDEPDC1B group were verified after sacrificing mice and removing xenografts (p < 0.01, Fig. 3C). Moreover, Ki-67 expression was significantly decreased in the shDEPDC1B group compared with the shCtrl group (Fig. 3D), indicating that DEPDC1B knockdown attenuated the proliferative ability of tumor cells in mice. Altogether, in vivo experiments were consistent with our previous findings, showing that DEPDC1B knockdown inhibited the ability of ESCC cells to form tumors.

### GABRD as a target protein of DEPDC1B in ESCC cells

**Fig. 4:**
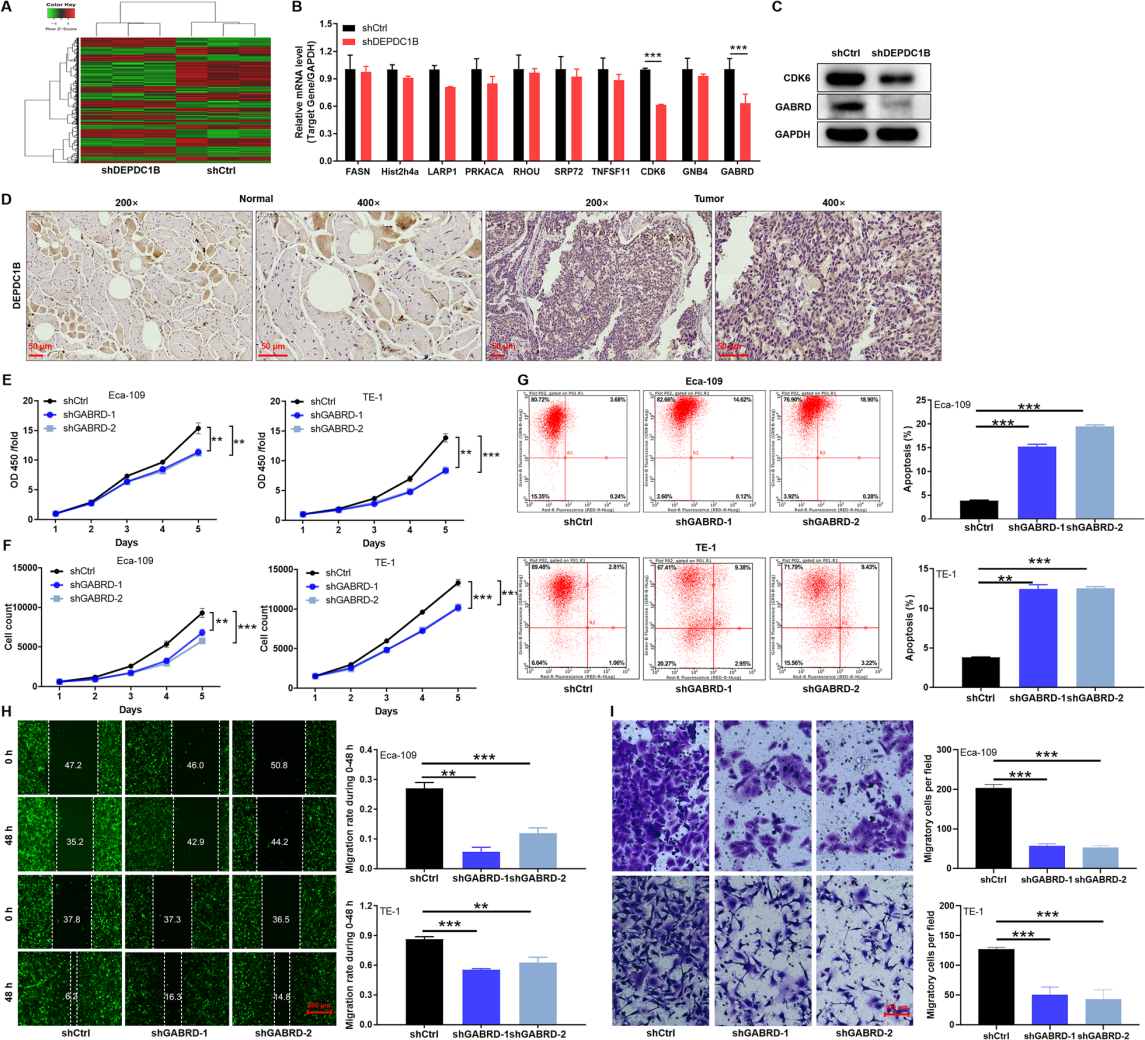
GABRD as a target protein of DEPDC1B in ESCC cells. **A** The heatmap of DEGs identified by microarray of cells treated with shCtrl (n = 3) or shDEPDC1B (n = 3). **B**, **C** The top DEGs with the highest ranking were selected and further validated by qRT-PCR (**B**) and western blotting (**C**). **D** GABRD expression was evaluated in ESCC tissues and normal tissues by IHC. **E** The effects of GABRD knockdown on Eca-109 and TE-1 cell proliferation in MTT assay. **F** Cell counting assays to assess the effect of GABRD knockdown on proliferation in Eca-109 and TE-1 cells. **G** The cell apoptosis was examined by flow cytometry upon GABRD knockdown. **H**, **I** wound-healing assay (H) and transwell assay (I) were used for determining migration of Eca-109 and TE-1 cells in shCtrl and shGABRD groups. The representative images were selected from 3 independent experiments, of which 5 fields were randomly selected for counting the cell numbers. **P < 0.01, ***P < 0.001

To further explore the potential mechanism by which DEPDC1B regulated ESCC, RNA sequencing was performed on Eca-109 cells (shCtrl vs shDEPDC1B, n = 3). The results indicated that knockdown of DEPDC1B resulted in a large number of downstream differentially expressed genes (DEGs). According to the screening criteria (log2 fold change > 1.3 and FDR < 0.05), downregulation of DEPDC1B induced upregulation of 461 genes and downregulation of 514 genes (Fig. 4A). Subsequently, the top DEGs with the highest ranking were selected and further validated by qRT-PCR (Fig. 4B). Moreover, knockdown of DEPDC1B resulted in a stronger downregulation of GABRD than CDK6 (Fig. 4C, Additional file 1: Fig. S3A), suggested that GABRD may act as a target protein of DEPDC1B. In addition, the expression level of GABRD in ESCC was verified based on the tissue microarrays from clinical ESCC patients using immunohistochemical experiments. The signal intensity of GABRD in tumor tissue is stronger than that in normal tissue (Fig. 4D). In addition, we constructed two shRNA-targeting GABRD sequences (shGABRD-1/2) (Additional file 1: Fig. S2A, B) and performed a series of functional validations in Eca-109 and TE-1 cells. Not surprisingly, knockdown of GABRD inhibited the malignant phenotypes of ESCC cells, which was manifested as inhibition of cell viability and proliferation (p < 0.01, Fig. 4E, F), enhanced apoptosis rate (p < 0.01, Fig. 4G), and impaired migration (p < 0.01, Fig. 4H, I). Altogether, GABRD, as a target protein of DEPDC1B, was required for ESCC cell proliferation and migration.

### Knockdown of GABRD alleviates the promotion of DEPDC1B on malignant progression of ESCC

**Fig. 5:**
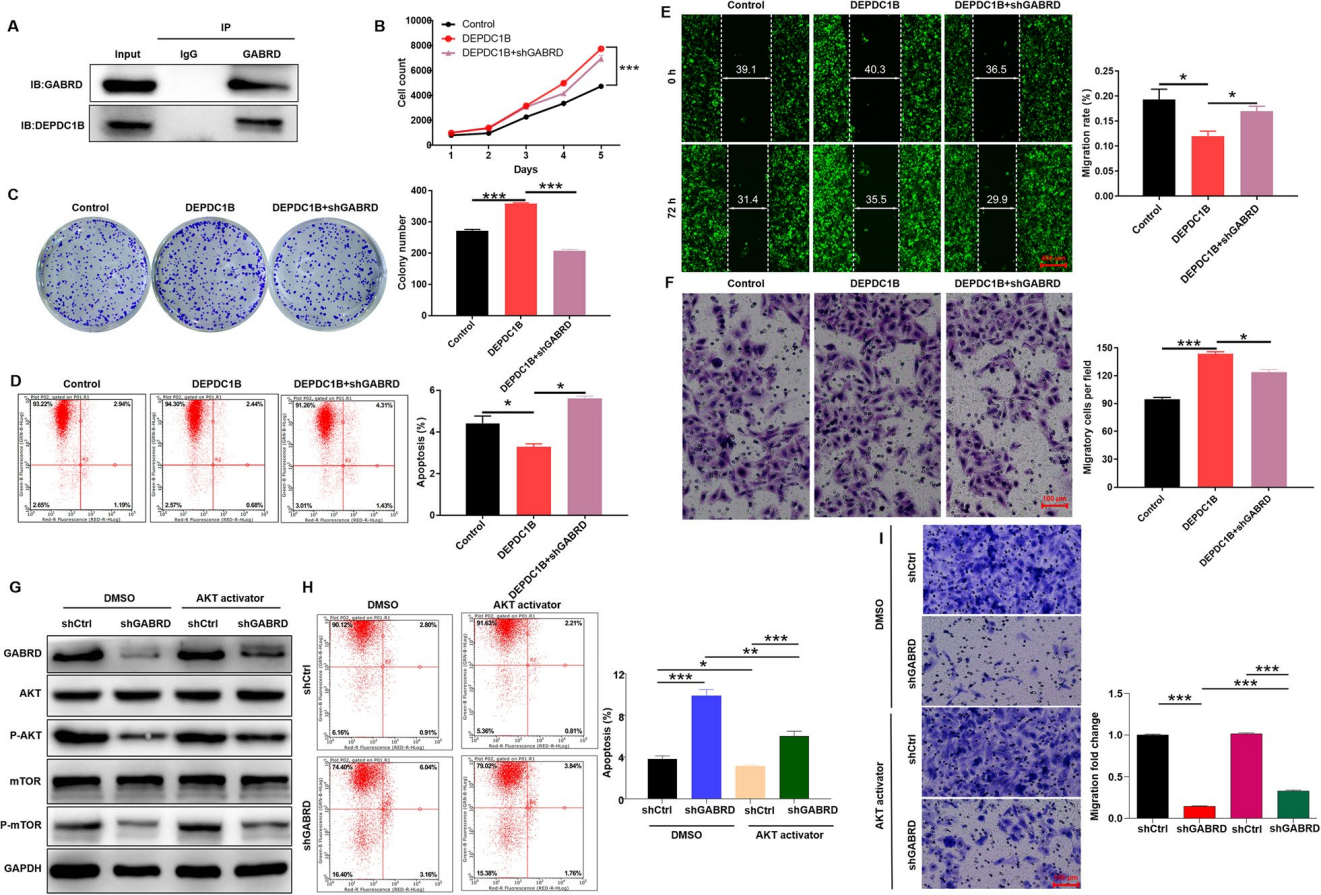
Knockdown of GABRD alleviates the promotion of DEPDC1B on malignant progression of ESCC. **A** The interaction of DEPDC1B and GABRD in Eca-109 cells were examined by Co-IP experiment. **B**–**F** GABRD-knockdown cells and DEPDC1B-overexpressing were constructed to conduct functional recovery experiments to determine the coordinated effect on proliferation (**B**, **C**), apoptosis (**D**) and migration (**E**–**F**). **G** Expression of canonical components of the PI3K/AKT/mTOR signaling pathway was examined in GABRD knockdown Eca-109 cells treated with AKT activator. **H–I** Apoptosis (**H**) and migration (**I**) ability of GABRD knockdown Eca-109 cells treated with AKT activator were examined. The representative images were selected from 3 independent experiments, of which 5 fields were randomly selected for counting the cell numbers. *P < 0.05, ***P < 0.001

To further clarify whether there was an interaction between DEPDC1B and GABRD, we performed Co-IP experiments. GABRD antibody can immunoprecipitated DEPDC1B protein, suggesting that DEPDC1B interacted with GABRD (Fig. 5A, Additional file 1: S3B). Furthermore, GABRD-knockdown cells (Additional file 1: Fig. S2A–D) and DEPDC1B-overexpressing (Additional file 1: Fig. S2E–G) were constructed to conduct functional recovery experiments to determine whether there is a coordinated effect between the two targets. As showed in Fig. 5B–F, DEPDC1B overexpression accelerates the malignant progression of ESCC, such as enhanced cell viability, colony forming and migratory abilities, and attenuated apoptosis. Interestingly, GABRD knockdown partially reversed the contribution of DEPDC1B to ESCC progression. Taken together, DEPDC1B and GABRD played a synergistic regulatory role in ESCC.

### GABRD regulates the progression of ESCC via PI3K/AKT/mTOR signaling pathway

As we all known, PI3K/AKT/mTOR is a typical cancer-promoting signaling pathway [24]. Knockdown of GABRD reduced the phosphorylation levels of AKT and mTOR, which was partially reversed by the addition of AKT activator (Fig. 5G, Additional file 1: S3C). Functionally, knockdown of GABRD enhanced ESCC cell apoptosis and inhibited migration, which could be alleviated by the addition of AKT activator (Fig. 5H, I). These results suggested that GABRD may be involved in the regulation of ESCC through the PI3K/AKT/mTOR signaling pathway.

## Discussion

ESCC is one of the most malignant cancers due to its advanced grade diagnosis, easy for metastasis, chemotherapy resistance [3]. There are still some challenges to overcome in the treatment of ESCC. Fortunately, during the past decade, the targeted therapy attracts more and more attentions from researchers and clinicians. Given that DEPDC1B plays a key role in multiple cancers, the role of this molecule in ESCC was explored to identify potential targets for ESCC patients. Firstly, the expression level of DEPDC1B in ESCC was revealed based on the TCGA database and immunohistochemical experiments on clinical tissues, identifying that DEPDC1B was overexpressed in ESCC. Moreover, the correlation between DEPDC1B and survival of ESCC patients was analyzed by Kaplan–Meier method. Our data indicated that high expression of DEPDC1B was significantly negatively correlated with overall survival in patients with ESCC. The results suggested that DEPDC1B may serve as a possible diagnostic and prognostic marker. Recently, Lai et al., reported that DEPDC1B was identified as a key regulator of bladder cancer development and could be used as a potential therapeutic target for bladder cancer therapy [17]. Moreover, Fan et al. indicated that DEPDC1B as an independent early diagnostic and prognostic biomarker for hepatocellular carcinoma [25]. Therefore, DEPDC1B may be a key regulator of cancer progression, and the detection of its expression level had important clinical value for diagnosis and prognosis of patients.

In addition, accumulating evidence demonstrated that DEPDC1B plays a key regulatory role in the progression of multiple cancers, including non-small-cell lung [12], hepatocellular [26], and bladder cancers [17], etc. For examples, silenced DEPDC1B could inhibited cell proliferation and induced cell apoptosis in malignant melanoma which is in agreement with our findings [19]. Consistently, DEPDC1B was able to promote migration and invasion of pancreatic cancer cell through Rac1/PAK1-LIMK1-Cofilin1 pathway [27]. In this study, shRNA-mediated silencing of DEPDC1B expression in ESCC cells and performed a series of in vitro and in vivo functional validations. We found that knockdown of DEPDC1B inhibited ESCC cell proliferation, clone formation, migration, tumor formation and promoted apoptosis.

Furthermore, knockdown of DEPDC1B leaded to significant downregulation of GABRD in ESCC cells. GABRD was identified as a downstream target of DEPDC1B. Gamma-aminobutyric acid type A receptor subunit delta (GABRD) has been suggested as a susceptibility gene to childhood-onset mood disorders and generalized epilepsies [28, 29]. Several recent studies have revealed a possible functional role of GABRD in tumors. Gross et al. found that GABRD was overexpressed in nearly 90% of cancer patients by using TCGA data analysis [30]. In addition, GABRD expression is significantly associated with poor prognosis in patients with colorectal cancer and colon adenocarcinoma [31, 32]. In this study, our data demonstrated that GABRD expression was upregulated in ESCC, and its silencing can inhibit the proliferation and migration of the tumor cells. Meanwhile, there was a protein interaction between DEPDC1B and GABRD. Functionally, GABRD knockdown partially reversed the contribution of DEPDC1B to ESCC progression. Nonetheless, the specific molecular mechanism between DEPDC1B and GABRD remained a limitation of our study, which required us to further explore.

As we all known, PI3K/AKT/mTOR is a typical cancer-promoting signaling pathway [24]. Related study demonstrated that GABA activated the PI3K-AKT signaling pathways that convey GABA signals responsible for β-cell proliferation and survival [33]. Besides, co-activation of GABA A receptor and GABA B receptor exerted neuroprotective effect via PI3K/AKT pathway [34]. The present study indicated that knockdown of GABRD reduced the phosphorylation levels of AKT and mTOR, which was partially reversed by the addition of AKT activator. Functionally, knockdown of GABRD enhanced ESCC cell apoptosis and inhibited migration, which could be alleviated by the addition of AKT activator. These results suggested that GABRD may be involved in the regulation of ESCC through the PI3K/AKT/mTOR signaling pathway. Thus, GABRD regulated ESCC progression may depend on PI3K/AKT/mTOR signaling pathway. However, the specific mechanism by which DEPDC1B/GABRD regulated ESCC via PI3K/AKT/mTOR signaling pathway required more studies to elucidate.

## Conclusion

Clinically, DEPDC1B expression was upregulated in ESCC and negatively correlated with overall survival of ESCC patients, which was of great value as a marker for early diagnosis and prognosis of these patients. Functionally, knockdown of DEPDC1B inhibited ESCC cell proliferation, clone formation, migration, tumor formation and promoted apoptosis. Meanwhile, GABRD knockdown partially reversed the contribution of DEPDC1B to ESCC progression. In conclusion, DEPDC1B cooperated with GABRD to regulate ESCC progression, and inhibition of this signaling axis may be a potential therapeutic target for ESCC.

## Supplementary Information

**Additional file 1.** Original data.

## Data Availability

All data generated or analyzed during this study are included in this published article and its Additional file 1.
