# Supplementary material for: DEPDC1B collaborates with GABRD to regulate ESCC progression
Source: Cancer Cell Int. 2022 Jun 15;22:214. doi: 10.1186/s12935-022-02593-z (PMC9202211; doi:10.1186/s12935-022-02593-z)
Supplement: Supplementary file 1 — Additional file 1. Original data. [file 12935_2022_2593_MOESM1_ESM.zip › σ╝áΘ£ôσÄƒσoïμò░μì«-2022-06-01/Figure 5H/shCtrl+AKT-2.pdf]

Well Number: D06 Sample ID: D06
File Name: E:/CWW/20210311 109 GRABD 激活剂 DW/2021-03-11_at_06-43-25pm.fcs

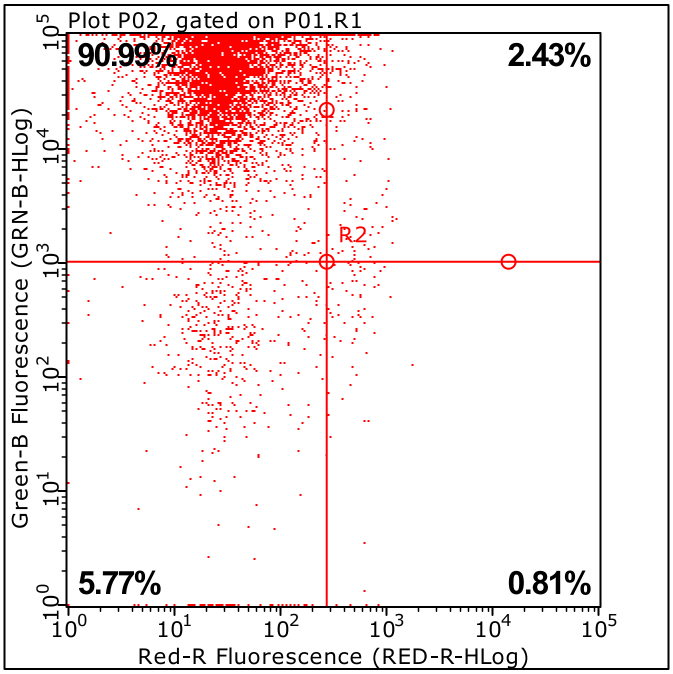


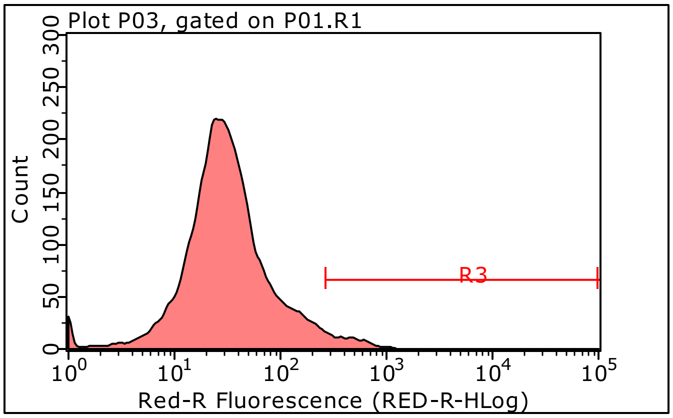

| Well | Sample ID | Date | R2.Percent.UL Percent for R2 gated by P01.R1 (%) | R2.Percent.UR Percent for R2 gated by P01.R1 (%) | R2.Percent.LL Percent for R2 gated by P01.R1 (%) | R2.Percent.LR Percent for R2 gated by P01.R1 (%) |
|---|---|---|---|---|---|---|
| D06 | D06 | 03.11.2021 | 90.99 | 2.43 | 5.77 | 0.81 |

| Well | R3.Percent Percent for R3 gated by P01.R1 (%) |
|---|---|
| D06 | 3.24 |
