# Supplementary material for: DEPDC1B collaborates with GABRD to regulate ESCC progression
Source: Cancer Cell Int. 2022 Jun 15;22:214. doi: 10.1186/s12935-022-02593-z (PMC9202211; doi:10.1186/s12935-022-02593-z)
Supplement: Supplementary file 1 — Additional file 1. Original data. [file 12935_2022_2593_MOESM1_ESM.zip › σ╝áΘ£ôσÄƒσoïμò░μì«-2022-06-01/Figure 5H/shCtrl+AKT-3.pdf]

Well Number: D07 Sample ID: D07
File Name: E:/CWW/20210311 109 GRABD 激活剂 DW/2021-03-11_at_06-43-25pm.fcs

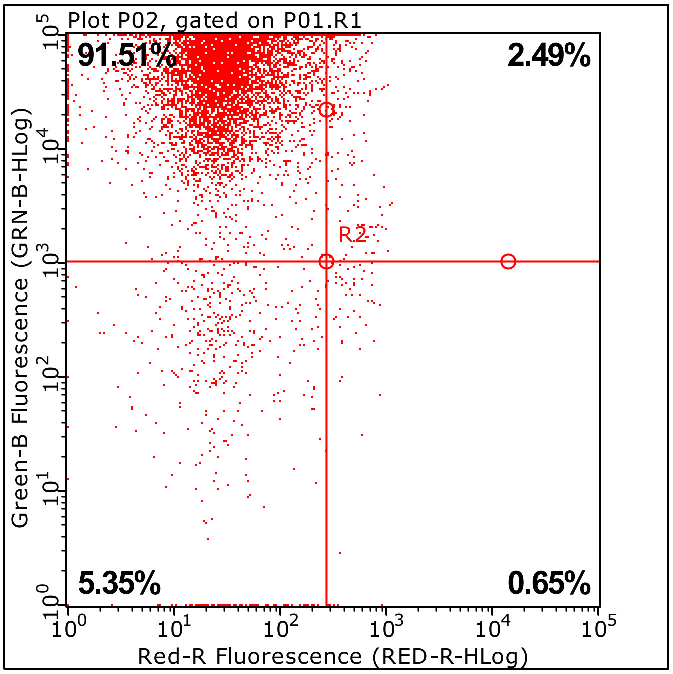

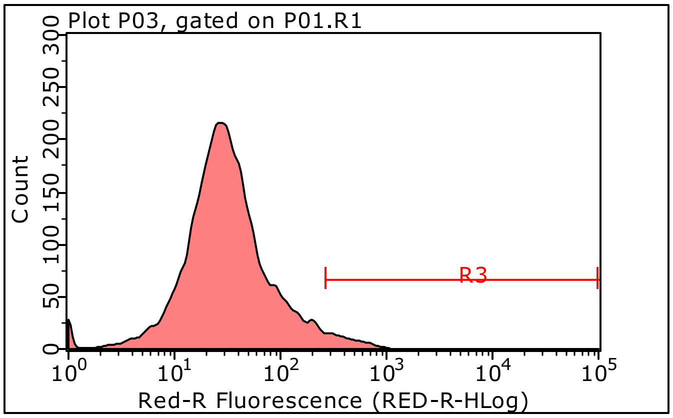

Well	Sample ID	Date	R2.Percent.UL Percent for R2 gated by P01.R1 (%)	R2.Percent.UR Percent for R2 gated by P01.R1 (%)	R2.Percent.LL Percent for R2 gated by P01.R1 (%)	R2.Percent.LR Percent for R2 gated by P01.R1 (%)
D07	D07	03.11.2021	91.51	2.49	5.35	0.65

Well	R3.Percent Percent for R3 gated by P01.R1 (%)
D07	3.11
