# Supplementary material for: DEPDC1B collaborates with GABRD to regulate ESCC progression
Source: Cancer Cell Int. 2022 Jun 15;22:214. doi: 10.1186/s12935-022-02593-z (PMC9202211; doi:10.1186/s12935-022-02593-z)
Supplement: Supplementary file 1 — Additional file 1. Original data. [file 12935_2022_2593_MOESM1_ESM.zip › σ╝áΘ£ôσÄƒσoïμò░μì«-2022-06-01/Figure 5D/DEPDC1B+shGABRD-3.pdf]

Well Number: C03 Sample ID: C03
File Name: C:/Users/ybr0067/Desktop/20200421 eca109 dw/2020-04-21_at_04-34-09pm.fcs

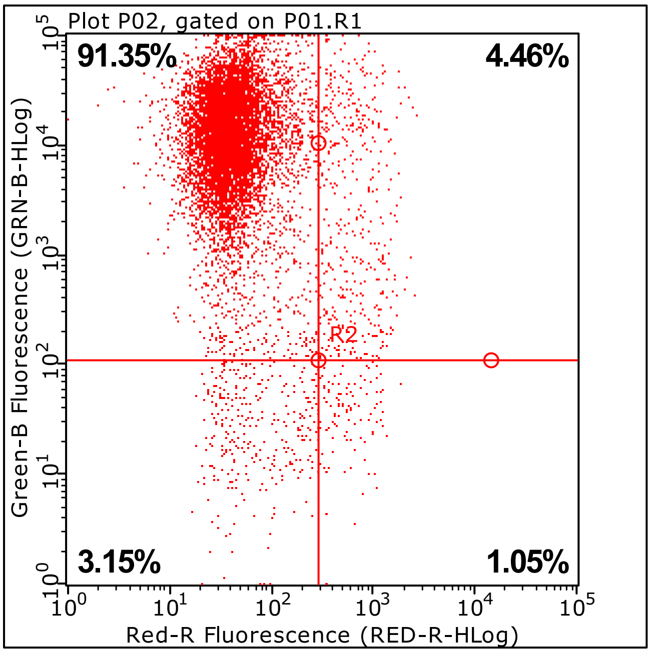


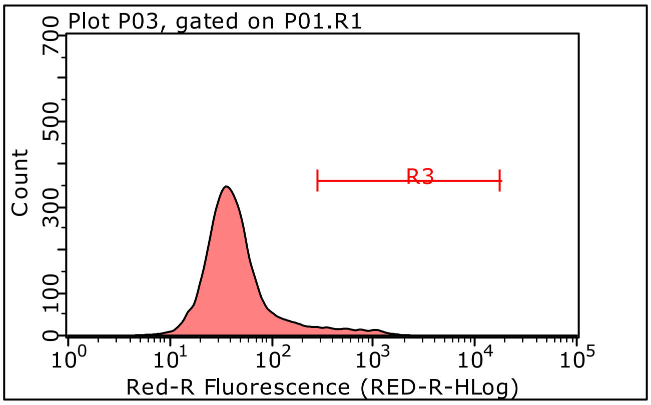

| Well | Sample ID | Date | R2.Percent.UL Percent for R2 gated by P01.R1 (%) | R2.Percent.UR Percent for R2 gated by P01.R1 (%) | R2.Percent.LL Percent for R2 gated by P01.R1 (%) |
|---|---|---|---|---|---|
| C03 | C03 | 04.22.2020 | 91.35 | 4.46 | 3.15 |

| Well | R2.Percent.LR Percent for R2 gated by P01.R1 (%) | R3.Percent Percent for R3 gated by P01.R1 (%) |
|---|---|---|
| C03 | 1.05 | 5.50 |
