# Supplementary material for: DEPDC1B collaborates with GABRD to regulate ESCC progression
Source: Cancer Cell Int. 2022 Jun 15;22:214. doi: 10.1186/s12935-022-02593-z (PMC9202211; doi:10.1186/s12935-022-02593-z)
Supplement: Supplementary file 1 — Additional file 1. Original data. [file 12935_2022_2593_MOESM1_ESM.zip › σ╝áΘ£ôσÄƒσoïμò░μì«-2022-06-01/Figure 2E/Eca-109/shDEPDC1B-2.pdf]

Well Number: C03 Sample ID: C03
File Name: C:/Users/dell/Desktop/lj-1/20180630-DW/2018-06-30_at_05-43-37pm-DEPDC1B-2.fcs

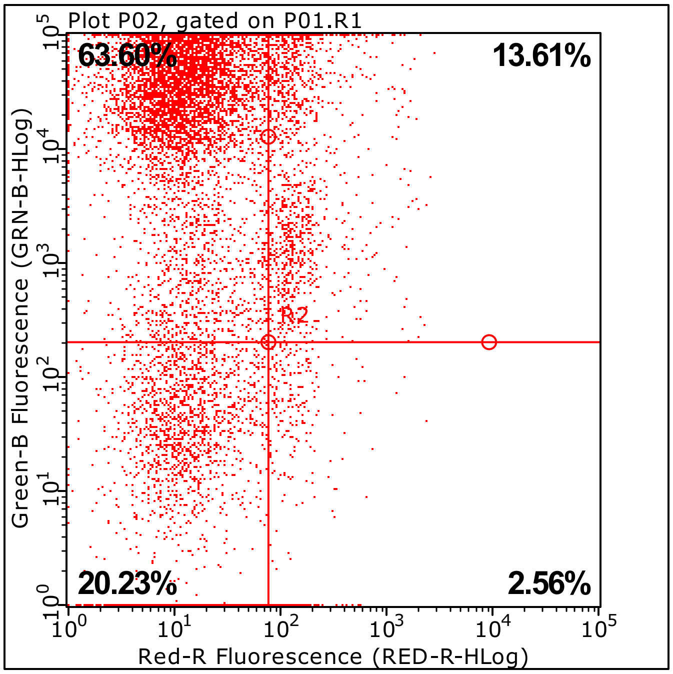

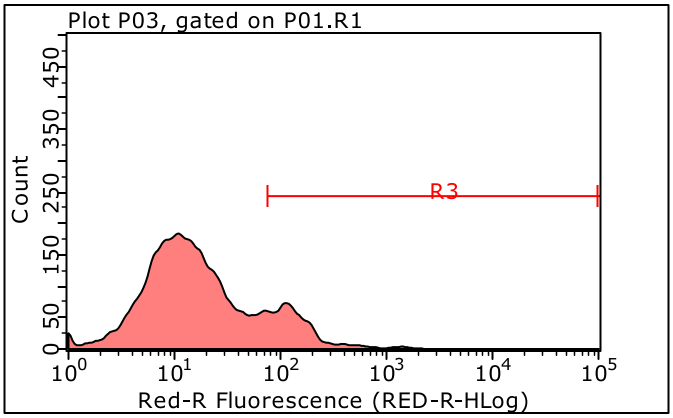

Well	Sample ID	Date	R2.Percent.UL Percent for R2 gated by P01.R1 (%)	R2.Percent.UR Percent for R2 gated by P01.R1 (%)	R2.Percent.LL Percent for R2 gated by P01.R1 (%)	R2.Percent.LR Percent for R2 gated by P01.R1 (%)
C03	C03	07.05.2018	63.60	13.61	20.23	2.56

Well	R3.Percent Percent for R3 gated by P01.R1 (%)
C03	16.17
