# Supplementary material for: DEPDC1B collaborates with GABRD to regulate ESCC progression
Source: Cancer Cell Int. 2022 Jun 15;22:214. doi: 10.1186/s12935-022-02593-z (PMC9202211; doi:10.1186/s12935-022-02593-z)
Supplement: Supplementary file 1 — Additional file 1. Original data. [file 12935_2022_2593_MOESM1_ESM.zip › σ╝áΘ£ôσÄƒσoïμò░μì«-2022-06-01/Figure 2E/TE-1/shDEPDC1B-3.pdf]

Well Number: C11
Sample ID: C11
File Name: E:/李杰/2018-07-18_at_01-06-28pm-TE-1-DEPDC1B.fcs

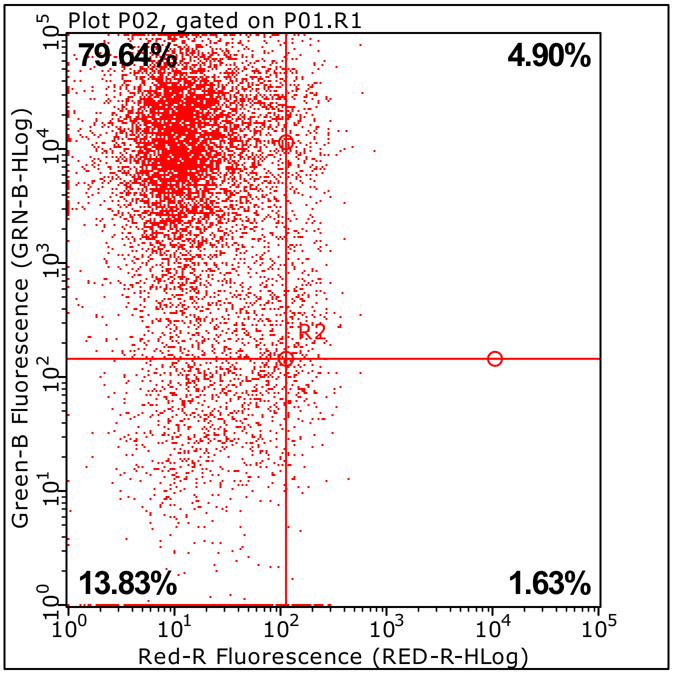

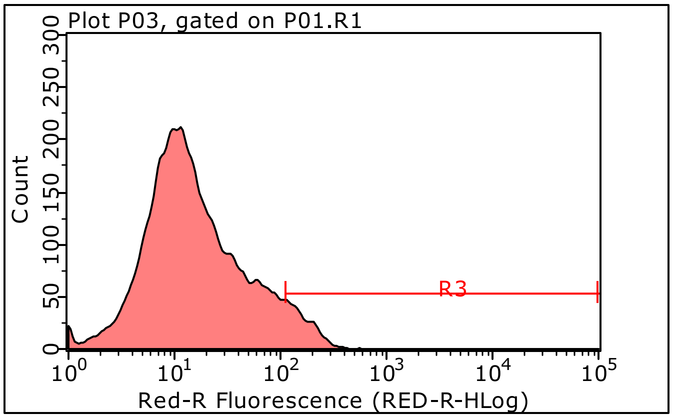

Well	Sample ID	Date	R2.Percent.UL Percent for R2 gated by P01.R1 (%)	R2.Percent.UR Percent for R2 gated by P01.R1 (%)	R2.Percent.LL Percent for R2 gated by P01.R1 (%)	R2.Percent.LR Percent for R2 gated by P01.R1 (%)
C11	C11	07.18.2018	79.64	4.90	13.83	1.63

Well	R3.Percent Percent for R3 gated by P01.R1 (%)
C11	6.53
