# Supplementary material for: DEPDC1B collaborates with GABRD to regulate ESCC progression
Source: Cancer Cell Int. 2022 Jun 15;22:214. doi: 10.1186/s12935-022-02593-z (PMC9202211; doi:10.1186/s12935-022-02593-z)
Supplement: Supplementary file 1 — Additional file 1. Original data. [file 12935_2022_2593_MOESM1_ESM.zip › σ╝áΘ£ôσÄƒσoïμò░μì«-2022-06-01/Figure 4G/Eca-109/shCtrl-2.pdf]

Well Number: F11
File Name: E:/tp/2019 04/2019 04 08 RKO DW/2019-04-08_at_06-04-14pm.fcs
Sample ID: F11

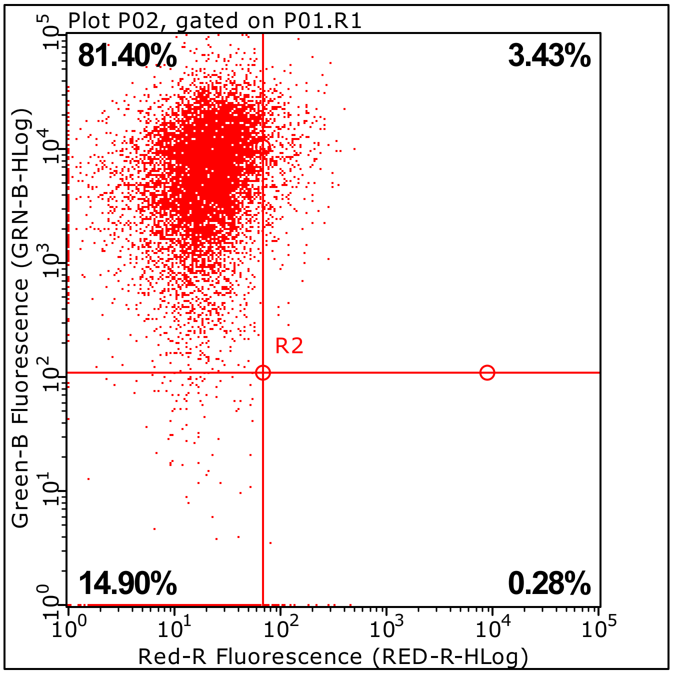

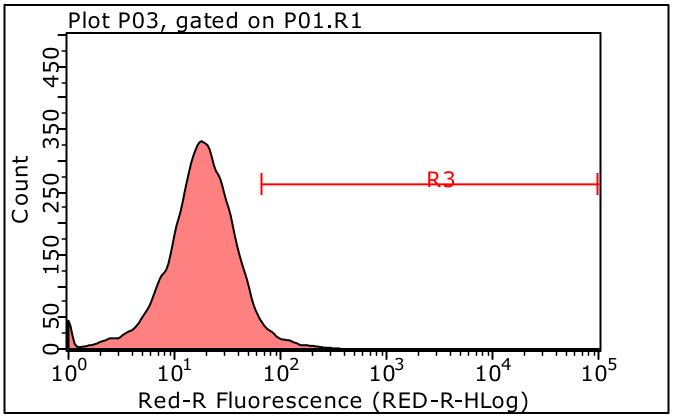

Well	Sample ID	Date	R2.Percent.UL Percent for R2 gated by P01.R1 (%)	R2.Percent.UR Percent for R2 gated by P01.R1 (%)	R2.Percent.LL Percent for R2 gated by P01.R1 (%)	R2.Percent.LR Percent for R2 gated by P01.R1 (%)
F11	F11	04.08.2019	81.40	3.43	14.90	0.28

Well	R3.Percent Percent for R3 gated by P01.R1 (%)
F11	3.71
