# Supplementary material for: DEPDC1B collaborates with GABRD to regulate ESCC progression
Source: Cancer Cell Int. 2022 Jun 15;22:214. doi: 10.1186/s12935-022-02593-z (PMC9202211; doi:10.1186/s12935-022-02593-z)
Supplement: Supplementary file 1 — Additional file 1. Original data. [file 12935_2022_2593_MOESM1_ESM.zip › σ╝áΘ£ôσÄƒσoïμò░μì«-2022-06-01/Figure 4G/TE-1/shGABRD-1-1.pdf]

Well Number: G06 Sample ID: G06
File Name: //192.168.1.121/正在处理/华北套餐组 (沈彤彤 陈若男 田东梅 刘梦 李婷婷 冉运聪 李光辉)/03 田东梅/仪器数据/流式/20210609 RKO X

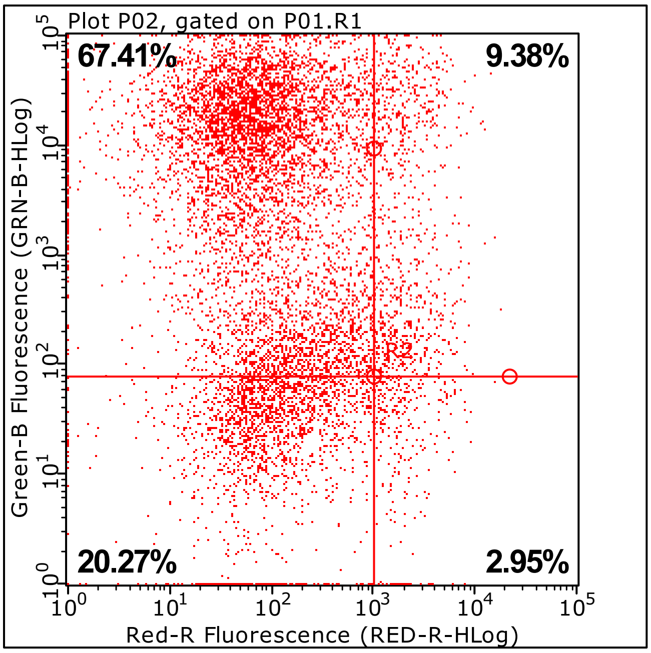

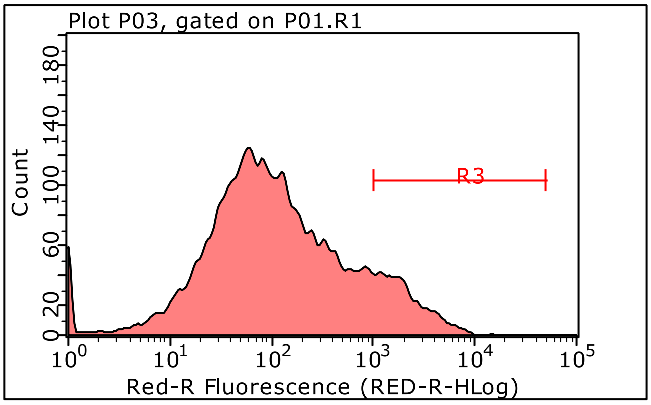

Well	Sample ID	Date	R2.Percent.UL Percent for R2 gated by P01.R1 (%)	R2.Percent.UR Percent for R2 gated by P01.R1 (%)	R2.Percent.LL Percent for R2 gated by P01.R1 (%)
G06	G06	06.12.2021	67.41	9.38	20.27

Well	R2.Percent.LR Percent for R2 gated by P01.R1 (%)	R3.Percent Percent for R3 gated by P01.R1 (%)
G06	2.95	12.24
